# Infiltration of Alternatively Activated Macrophages in Cancer Tissue Is Associated with MDSC and Th2 Polarization in Patients with Esophageal Cancer

**DOI:** 10.1371/journal.pone.0104453

**Published:** 2014-08-21

**Authors:** Jingjing Gao, Yumin Wu, Zhaoliang Su, Prince Amoah Barnie, Zhijun Jiao, Qingli Bie, Liwei Lu, Shengjun Wang, Huaxi Xu

**Affiliations:** 1 Department of Immunology, School of Medical Science and Laboratory Medicine, Jiangsu University, Zhenjiang, P. R. China; 2 Department of Laboratory Medicine, Affiliated Hospital of Jiangsu University, Zhenjiang, P. R. China; 3 Department of Laboratory Medicine, Suzhou Hospital Affiliated to Nanjing Medical University, Suzhou, P. R. China; 4 Department of Pathology and Centre of Infection and Immunology, The University of Hong Kong, Hong Kong, P. R. China; Centre de Recherche Public de la Santé (CRP-Santé), Luxembourg

## Abstract

Myeloid derived suppressor cells (MDSCs) expand in cancer bearing hosts and contribute to tumor immune evasion. M2 macrophages constitute a major cellular component of cancer-related inflammation. However, the correlation between circulating MDSCs and infiltrating M2 macrophages in tumor tissues from patients with esophageal cancer (ECA), and its potential relationship with the polarization of Th2 cells remain unclear. In the present study, we showed the level of MDSCs in PBMC and Arg1 in plasma were significantly elevated in ECA patients, and the increased ratio of MDSC in PBMC was closely related to the expression of CD163 in cancer tissues. In addition, the ECA patients exhibited remarkable increases in the mRNA levels of IL-4 and GATA3, as well as the protein levels of IL-13 and IL-6, but IFN-γ and IL-12 in peripheral blood were decreased. Our data indicate that the increased Th2 cytokines are associated with MDSCs and M2 macrophages polarization, and foster the infiltration of CD163^+^M2 macrophages in cancer tissues, which promote the formation of immunosuppressive microenvironment in ECA patients.

## Introduction

Esophageal cancer (ECA) is one of the most common malignancies all over the world and the fourth leading cancer death in China [1]–[3]. There are increasing evidences of the crucial role of the immune system in malignant tumors, but the precise mechanisms of immune modulation in ECA patients remain elusive. In the past decade, the role of the immunosuppressive and cancer-promoting myelomonocytic compartment within the tumor environment has also received a great deal of attention [4]. Although mechanisms are incompletely understood, cancer promotes the accumulation of a heterogeneous pool of bone marrow-derived immature, poorly differentiated myelomonocytic cells (monocytes, neutrophils, immature macrophages and dendritic cells), called myeloid derived suppressor cells (MDSCs), which are major host component contributing to the immunosuppressive environment. In pathological conditions, a partial block in the differentiation of immature myeloid cells into matured myeloid cells results in an expansion of MDSCs [5]. Recently, it has become clear that the suppressive activity of MDSCs requires various factors which promote their expansion or induce their activation. These factors, which include IL-4, IL-13, ligands for Toll-like receptors (TLRs), and transforming growth factor-β (TGF-β), activate several different signalling pathways in MDSCs that involve STAT6 and nuclear factor-κB (NF-κB).

Similar to MDSCs, macrophages are a diverse population of myeloid cells which undergo specific differentiation depending on the stimulating agents, as documented extensively. Recent immunological studies have identified two distinct states of the polarized macrophage activation: the classically activated (or M1) and the alternatively activated (or M2) macrophage phenotypes. M1 macrophages are typically activated by IFN-γ and LPS, whereas M2 macrophages are activated by IL-4 and IL-13 [6]. M1 macrophages are tumoricidal and promote tumor rejection, whereas M2 macrophages promote tumor progression [7]–[9]. Both M2 macrophages and MDSCs are a major source of immunosuppression that allows tumor-escape from effective anti-cancer responses, but no information on the relationship between M2 macrophages and MDSCs in the development of esophageal cancer is currently available.

An exciting observation has been described in the laboratory of Dr Teramoto, whose group show that simultaneous activation of Th1 function can augment the potency of dendritic cell-based cancer immunotherapy [10]. In our previous study, we have found that there is a predominant Th2 phenotype in patients with gastric cancer [11]. It is conjectured that the imbalance of Th1/Th2 may contribute to the occurrence and development of tumor. MDSCs and/or M2 macrophages, as major host components contributing to the suppressive environment of tumor immunity [12], their polarization should be related to the immune balance disorders. In the current study, we analyzed the levels of M2 macrophages and MDSCs in ECA patients, detected the expression of some related factors including Th1/Th2 type cytokines and evaluated the relationship between Th2 cells and M2 macrophages or MDSCs. The general goal of the study was to contribute to better understanding of the significance of MDSCs and M2 macrophages from patients with esophageal cancer in Th2 cell polarization state.

## Materials and Methods

### Patients

Fifty newly diagnosed ECA patients receiving treatment at the Affiliated People’s Hospital of Jiangsu University were included in this study: 38 males and 12 females, with mean age 61.97±1.24 years. None of the patients had undergone radiotherapy or chemotherapy prior to the start of the current study. The characteristics of ECA patients were summarized in Table 1. All primary tumor cases were staged clinically according to the guidelines of the International Union for Cancer Control and American Joint Committee on Cancer (UICC-AJCC) updated the tumor- node-metastasis (TNM) cancer staging system [13]. Thirty healthy volunteers without any chronic inflammatory condition were studied simultaneously as control, comprising 24 males and 6 females. Enrollment took place between March 2011 and December 2012. This study was approved by the ethical committee of Jiangsu University, and written informed consent was obtained from all individuals.

**Table 1:** Characteristics of ECA patients enrolled for the study.

Characteristics	Early disease^a^ (n = 11)	Advanced disease^a^ (n = 39)
Age		
>60	9	34
≤60	2	5
Sex		
Male	8	30
Female	3	9
Location		
Upper	2	7
Middle	6	24
Lower	3	8
Tumor size		
>3 cm	3	30
≤3 cm	8	9
Histological grading		
Well-G1	4	6
Moderate-G2	5	28
Poor-G3	2	5
TNM stage^b^		
I	11	
IIA		10
IIB		14
III		9
IV		6

a Esophageal cancer with early disease corresponds to stage I and those with advanced disease correspond to stages II, III, and IV.

b Stage: according to the TNM classification for esophageal cancer (UICC, 2010).

### Cell preparation

Peripheral blood samples were collected from ECA patients and healthy volunteers. Peripheral blood mononuclear cells (PBMCs) were isolated by Ficoll-Hypaque density-gradient centrifugation (GE Healthcare). The cell suspensions were divided into two equal aliquots; one was immediately used for the experiments. The other was cryopreserved at −80°C and thawed for testing at a later date.

### Flow cytometry

MDSCs population was defined as HLA-DR^−^/CD14^−^/CD11b^+^/CD33^+^. Heparinized venous blood was freshly obtained from ECA patients or healthy volunteers. PBMCs were isolated by standard Ficoll-Hypaque density centrifugation, and stained with the following antibody mix: FITC-conjugated anti-human HLA-DR, PerCP-Cy5.5-conjugated anti-human CD14, APC-conjugated anti-human CD11b, or PE-conjugated anti-human CD33. The isotype control antibody was used in all cases. Data acquisition and analysis were performed on a FACSCalibur flow cytometer (Becton Dickinson, CA, USA) using CellQuest software (Becton Dickinson, CA, USA).

### RNA extraction and quantitative real-time PCR

Following the manufacturer’s instructions, total RNA was extracted from PBMCs using Trizol reagent (Invitrogen, Carlsbad, CA). Complementary DNA was synthesized from 1µg total RNA using RT reagent kit (TaKaRa, Ohtsu, Japan). For quantitative real-time PCR, reverse transcribed cDNA (2µl) was amplified by real-time PCR with the SYBR Green Premix EX Taq kit (TaKaRa, Ohtsu, Japan). Each sample was analyzed in duplicate with the CFX-96 Cycler (Thermal) and the normalized signal level was calculated based on its ratio to the corresponding β-actin housekeeping signal. Primers used in PCR were showed in Table 2.

**Table 2:** Primers used in real-time PCR.

Gene	Upper/Lower	Sequence (5′-3′)	Length (bp)
β-actin	U	CACGAAACTACCTTCAACT	265
	L	CATACTCCTGCTTGCTGATC	
IFN-γ	U	TTGGGTTCTCTTGGCTGTTA	96
	L	ATCCGCTACATCTGAATGACCT	
T-bet	U	CGGGAGAACTTTGAGTCC	115
	L	ACTGGTTGGGTAGGAGAGGAG	
IL-4	U	GACATCTTTGCTGCCTC	99
	L	TACTCTGGTTGGCTTCCTTCA	
GATA3	U	AGACCACCACAACCACACT	122
	L	GATGCCTTCCTTCTTCATAGTCA	
IL-12	U	TTGCCTAAATTCCAGAGAGA	150
	L	AGCTTTGCATTCATGGTCTTG	
IL-13	U	ATCCTCTCCTGTTGGCAC	155
	L	CTGGTTCTGGGTGATGTTGAC	

IFN, Interferon; IL, Interleukin; U, Upper; L, Lower.

### Enzyme-linked immunosorbent assay (ELISA)

For quantitative detection of IFN-γ, IL-4, IL-6, IL-13 and Arg1 in plasma, commercially available ELISA kits were used according to the manufacturer’s instruction (Biovender). All samples were run in batches to minimize inter-assay variability and quantitated using a standard curve.

### Immunohistochemistry

ECA tissues and paired adjacent tissues were fixed in 10% buffered formalin solution and embedded in paraffin. Serial sections of 4 µm were cut from the paraffin blocks. CD68 or CD163 antibody was used respectively to identify macrophages and M2 macrophage subtype. As negative controls, the primary antibodies were replaced by an irrelevant isotype anti-mouse IgG1. Immunohistochemical detection was performed using the avidin-biotin-peroxidase method. A multiheaded microscope was used to read immunohistochemical results, the detected cells were countered at 10 randomly selected areas by three different researchers at ×40 magnification. The final result was estimated according to the cell color depth and the percentage of stained cells.

### Statistical analysis

The statistical analysis was performed with GraphPad Prism, Version 5.0, software (San Diego, CA, USA). Correlations between variables were determined by Spearman’s correlation coefficient. Comparisons between groups were performed using the Student’s unpaired or paired *t* test. Differences were considered statistically significant when the *P*-value <0.05.

## Results

### Elevated MDSCs correlated with increased plasma level of Arg1 in ECA patients

**Figure 1:**
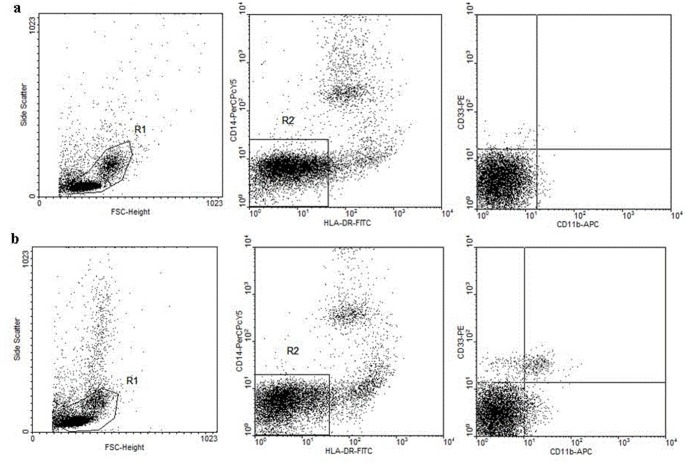
The frequency of circulating MDSCs (HLA-DR^−^/CD14^−^/CD11b^+^/CD33^+^ cells) increased in ECA patients. The frequency of MDSCs in PBMC was analyzed by flow cytometry, the patients and healthy controls used in this experiment had been matched with sex, age and number. a: Representative diagram of flow cytometry analysis for circulating MDSCs from healthy controls. b: Representative diagram of flow cytometry analysis for circulating MDSCs from ECA patients. MDSCs, myeloid-derived suppressor cells; ECA, esophageal cancer; FSC, forward scatter; FITC, fluorescein isothiocyanate; PerCP-Cy5.5, peridinin chlorophyll protein-cyanin 5.5; PE, phycoerythrin; APC, allophycocyanin.

MDSCs, defined as HLA-DR^−^/CD14^−^/CD11b^+^/CD33^+^ cells, were determined by flow cytometry and calculated as % PBMC. The level of individual MDSCs was significantly elevated in ECA patients compared to healthy controls (*P*<0.05; Figure 1). As shown in Table 3, the proportion of MDSCs in patients with advanced cancer was significantly higher than that in patients with early cancer (2.56±0.25% vs. 0.77±0.15%; *P*<0.05). Moreover, the proportion of MDSCs in patients with lymph node metastasis was higher than those without lymph node metastasis (3.71±0.34% vs. 1.47±0.13%; *P*<0.0001). However, there were no significant differences between the proportion of MDSCs and the patients’ age, gender, tumor location, tumor size, or tumor infiltrating depth. We also measured the plasma concentration of Arg1, which is one of the most important suppressive factors [14], [15]. The result showed that control plasma had minimal Arg1 compared to ECA patients (9.57±1.51 ng/ml vs. 28.28±4.10 ng/ml; *P*<0.001). In addition, a positive correlation was found between the proportion of MDSCs from PBMC and plasma level of Arg1 in ECA patients in subsequent tests.

**Table 3:** Relationship between % MDSCs and clinical pathological profile.

	n	MDSC%	P
Age			
>60	43	2.43±0.33	>0.05
≤60	7	1.78±0.27	
Sex			
male	38	2.38±0.29	>0.05
female	12	1.52±0.22	
Tumor size(cm)			
>3	33	2.36±0.32	>0.05
≤3	17	1.88±0.28	
Histological grading			
Well- G1	10	0.77±0.16	>0.05
Moderate- G2	33	1.96±0.12	
Poor- G3	7	4.43±0.28	
Tumor infiltrating depth			
T1+T2	15	1.92±0.22	>0.05
T3+T4	35	2.16±0.16	
Lymph node metastasis			
Yes	17	3.71±0.34	<0.0001
No	33	1.47±0.13	
TNM stage			
(Early cancer)	11	0.77±0.15	<0.05
II+III+IV (Advanced cancer)	39	2.56±0.25	

### Enhanced M2 macrophages in ECA patients

**Figure 2:**
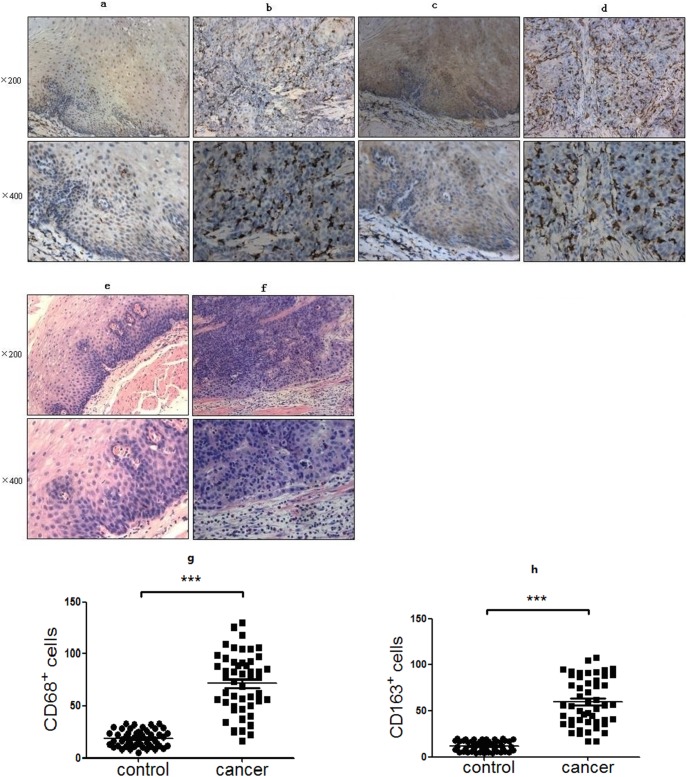
Immunohistochemical analysis of CD68 and CD163 expression in esophageal cancer and adjacent tissues. Representative immunohistochemical pictures showed the expression of CD68 in a cancer-adjacent tissue (a). and a cancer tissue (b). Representative immunohistochemical pictures showed the expression of CD163 in a cancer-adjacent tissue (c). and a cancer tissue (d). Normal esophageal squamous epithelium (haematoxylin-eosin/HE staining) (e). Squamous cell esophageal carcinoma (HE staining) (f). Analysis of the number of CD68^+^ macrophages (g). and CD163+ macrophages (h). in cancer and cancer-adjacent tissues, the results showed that most cancer tissues had larger number of CD68+ and CD163+ macrophages infiltration than that in the cancer-adjacent tissues.

CD163 is a scavenger receptor, upregulated by macrophages in anti-inflammatory environments [16] and regarded as a highly specific monocyte/macrophage marker for M2 macrophages [17]–[19]. Most papers in the meta-analysis used CD68 as a marker for tumor-associated macrophages (TAMs) [20]. CD68, however, recognizes both M1 and M2 macrophages [21]. Therefore, we used CD68 and CD163 as the markers of macrophages and M2 macrophages respectively. To analyze whether the localization of CD163^+^ and CD68^+^ macrophages had a correlation to clinical characteristics, the distribution of CD163^+^ and CD68^+^ macrophages in tumor and tumor-free tissues was evaluated separately (Figure 2). The staining categories were initially scored from 0 to 3 in macrophage infiltration density. Results showed that most tumor tissues had higher densities of CD163^+^ and CD68^+^ macrophages infiltration than those in the tumor-free tissues. The positive expression rates of CD163^+^ and CD68^+^ were 68% and 78% respectively in cancer tissues compared to 4% and 6% in the adjacent cancer tissues. Moreover, the increased ratio of MDSC in PBMC from ECA patients was closely related to the expression of CD163 in cancer tissues (Tables 4–5).

**Table 4:** Expression of CD68/CD163 in esophageal cancer tissues and adjacent tissue.

groups	n	CD68/CD163*			positi	*P*
		−	+	++	%	
adjacent tissue	50	47/48	3/2	0/0	6	<0.05
cancer tissues	50	11/16	13/14	26/20	78/68	

*Ten high magnification views were selected to count all cells, the cell color depth and the percentage of stained cells as the judgment result basis. According to the staining color (A): 0 = no color in cytoplasm; 1 = light yellow; 2 = pale brown; 3 = brown color. According to the percentage of stained cells (B): “0” indicates the percentage of positive cells <5%; “1″ indicates the percentage of positive cells 5%–25%; “2″ indicates the percentage of positive cells 26%–50%; “3” represents the percentage of positive cells >50%. The two scores of A plus B as a final judgment result: 0∼1: “−”; 2∼3: “+”; 4∼6: “++”.

**Table 5:** Relation between CD163 expression in cancer tissues and MDSC% in PBMC from ECA patients.

The number of patients	CD163 expression	MDSC% (x±s)	*P*
16	−	1.43±0.13	<0.0001
14	+	2.23±0.05	
20	++	3.55±0.15	

### Increased IL-4 and GATA3 mRNA while decreased IFN-γ mRNA in PBMC from ECA patients

**Figure 3:**
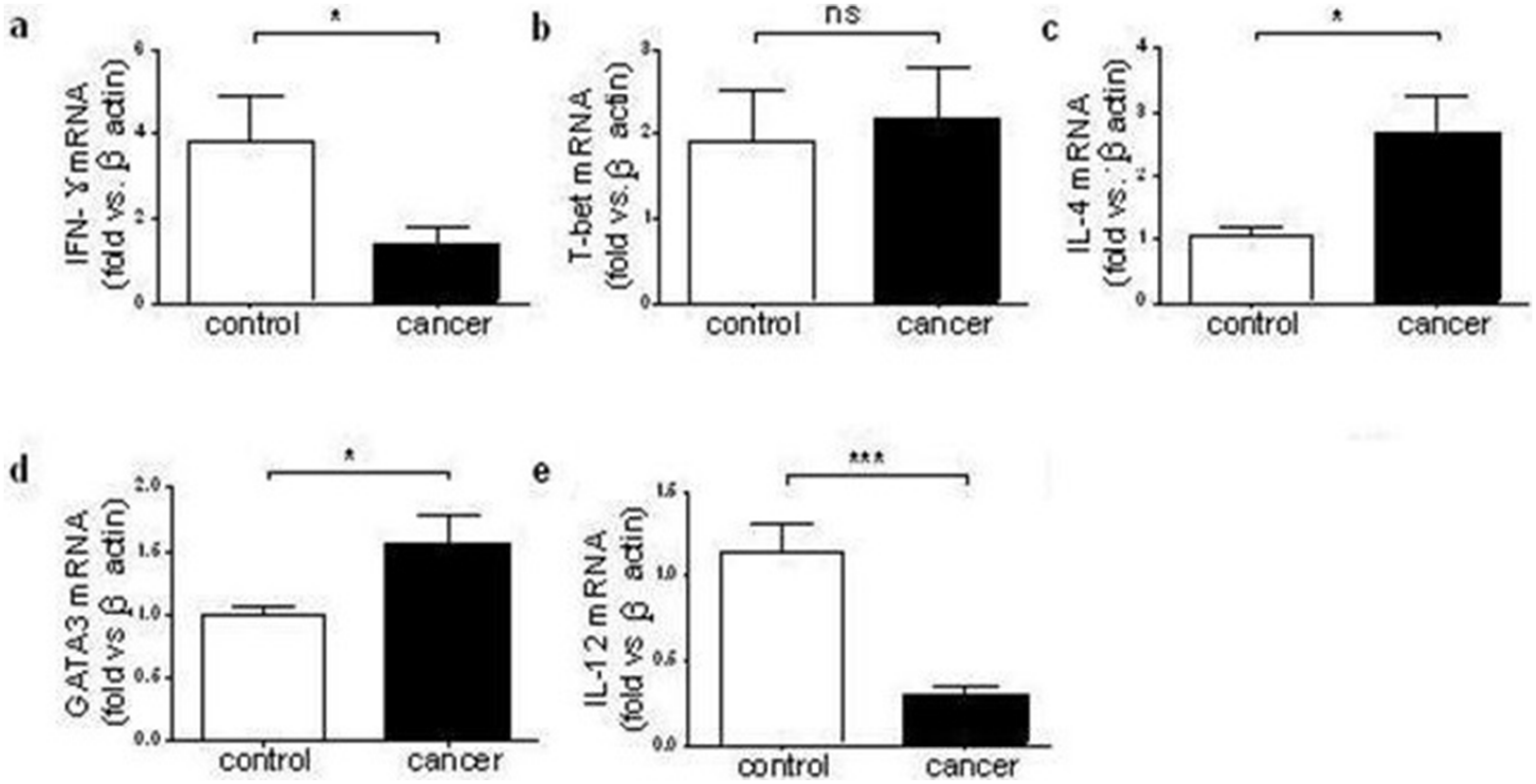
The mRNA level of each gene in PBMC from ECA patients and healthy controls. The mRNA levels of IFN-γ (a), T-bet (b), IL-4 (c), GATA3 (d) and IL-12 (e) were determined by real-time PCR. Data were analyzed by the Student’s t-test. *P<0.05, ***P<0.001 vs. control group. NS, no significant difference. ECA, esophageal cancer; PCR, polymerase chain reaction; IFN, interferon; IL, interleukin. The patients and healthy controls used in this experiment had been matched with sex, age and number.

The relative expression levels of cytokine genes encoding IL-4, IL-6, IL-12, IL-13 and IFN-γ, as well as transcription factors GATA3 and T-bet were assessed using real-time PCR. As shown in Figure 3, ECA patients had increased expressions of IL-4 and GATA3 mRNA compared to the healthy controls (*P*<0.05). IFN-γ, IL-12, and IL-13 mRNA expressions in ECA group were significantly lower than those in the control group (all *P*<0.05). But no significant difference was found in terms of T-bet mRNA expression between the two groups (*P*>0.05).

### Increased IL-6, IL-13, Arg1 in plasma from ECA patients

**Figure 4:**
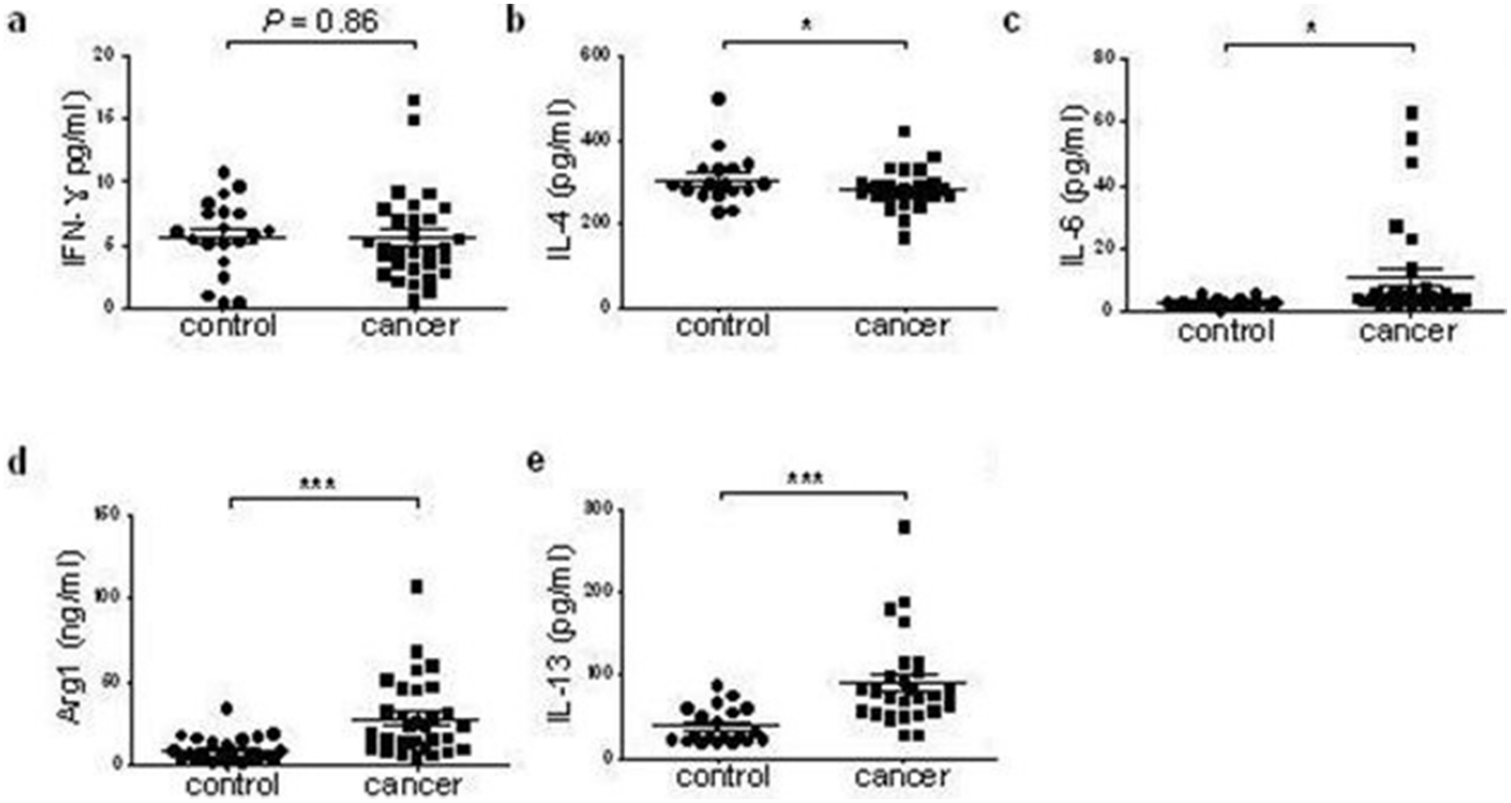
Plasma concentrations of cytokines in ECA patients and healthy controls. The patients and healthy controls used in this experiment had been matched with sex, age and number. Plasma concentrations of IFN-γ (a), IL-4 (b), IL-6 (c), Arg1 (d) and IL-13 (e) were determined by ELISA. Data were analyzed by the Student’s t-test. *P<0.05, ***P<0.001 vs. control group. NS: no significant difference, ECA: esophageal cancer, ELISA: enzyme linked immunosorbent assay, IFN: interferon, IL: interleukin, Arg1: arginase 1.

Plasma IFN-γ, IL-4, IL-6, IL-13, and Arg1 were determined by ELISA. As shown in Figure 4, IL-6 and IL-13 concentrations were significantly higher in the ECA patients compared to the control subjects (*P*<0.05 and *P*<0.001). In addition, a higher level of Arg1 was found in plasma from ECA patients. However, there were no significant differences in IFN-γ or IL-4 concentration between the two groups.

### Correlation analysis between different cytokines in ECA patients

**Figure 5:**
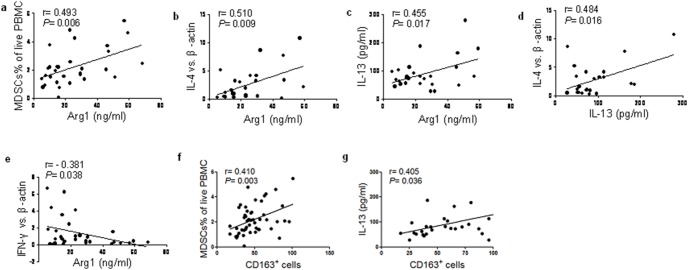
Correlation between cytokines and MDSCs as well as M2 macrophages in ECA patients. A: Correlation between the plasma concentration of Arg1 and the percentages of circulating MDSCs in ECA patients. Positive correlation was found between Arg1 and MDSCs (r = 0.493, *P* = 0.006). b: Correlation between the plasma concentration of Arg1 and the mRNA level of IL-4 from ECA patients. Positive correlation was found between Arg1 and IL-4 mRNA (r = 0.510, *P* = 0.009). c: Correlation between the plasma concentrations of Arg1 and IL-13 from ECA patients. Positive correlation was found between Arg1 and IL-13 (r = 0.455, *P* = 0.017). d: Correlation between the mRNA level of IL-4 and plasma level of IL-13 from ECA patients. Positive correlation was found between IL-4 and IL-13 (r = 0.484, *P* = 0.016). e: Correlation between the mRNA level of IFN-γ and plasma level of Arg1 from ECA patients. Negative correlation was found between IFN-γ and Arg1 in ECA patients (r = −0.381, *P* = 0.038). f: Correlation between the number of CD163^+^ macrophages in cancer tissues and the percentages of circulating MDSCs in PBMC from ECA patients (r = 0.410, *P* = 0.003). g: Correlation between the number of CD163^+^ macrophages in cancer tissues and the plasma concentration of IL-13 from ECA patients (r = 0.405, *P* = 0.036).

The correlations between the various cytokines in the ECA patients were analyzed. As shown in Figure 5, there was a significant positive correlation between the levels of Arg1 (MDSCs and M2 associated cytokine) and IL-13 (Th2 type cytokine) in plasma from the ECA patients. IL-4, as a cytokine of Th2, the mRNA expression was increased following the plasma Arg1 enhancement, but a negative correlation was found between IFN-γ and Arg1. In addition, the concentration of IL-13 in plasma showed positive correlation with the mRNA level of IL-4 in PBMC (r = 0.45, *P*<0.05).

## Discussion

MDSCs, under normal conditions migrate to different peripheral organs and differentiate into dendritic cells, macrophages and/or granulocytes. However, factors produced in the tumor microenvironment, alone or in combination, promote the accumulation of MDSCs, prevent their differentiation and induce their activation. In the tumor environment, MDSCs can also differentiate into TAMs, which are a unique phenotype whose function is distinct from MDSCs [5]. Macrophages are plastic cells, as they can switch from an activated M1 state back to an M2/TAMs state, and vice versa, upon the induction of specific signals. Macrophages infiltrating in cancer tissues are referred to as TAMs, which are closely involved in the development of the tumor microenvironment. Heterogeneity of phenotypes is observed among TAMs in various malignant tumors, and a significant proportion of TAMs/M2 phenotype is associated with a worse clinical prognosis and high grade of malignancy [22]. In 2007, Sinha et al. [23], used the spontaneously metastatic 4T1 mouse mammary carcinoma, and demonstrated that MDSCs impaired tumor immunity by suppressing T cell activation as well as interacting with macrophages to increase IL-10 and decrease IL-12 production, thereby promoting a tumor-promoting type 2 response, a process which can be partially reversed by gemcitabine. Based on this, our study targeted ECA and investigated MDSCs in peripheral blood, macrophages in the tumor tissues and the levels of some related factors. As expected, increased MDSCs were found in peripheral blood, and some amount of M2 macrophages infiltrated into the tumor sites, which indicated that these cell populations were involved in the pathogenesis of ECA. In addition, increased plasma level of Arg1, which was correlated with MDSCs and M2 macrophages functions, was also found in ECA patients. Notably, the increased ratio of MDSCs in PBMCs from ECA patients was closely related to the expression of CD163 in cancer tissues.

Previously we showed that there was a predominant Th2 phenotype in gastric cancer patients, and it was believed that the Th2 associated cytokines IL-4, IL-13, IL-6 and transcriptional factors GATA3 might closely be related to the polarization of M2 and MDSCs,and fostered an immunosuppressive environment in cancer patients. In the present study, we demonstrated that ECA patients exhibited remarkable increases in the mRNA levels of IL-4 and GATA3, as well as the plasma levels of IL-13 and IL-6. In contrast, plasma level of IFN-γ and mRNA levels of IFN-γ and IL-12 were decreased. There was a positive correlation between the mRNA level of IL-4 and plasma level of IL-13, and these two cytokines increased following the Arg1 enhancement in plasma. In the meantime, a negative correlation was found between IFN-γ and Arg1. The present data indicated that there was a predominant Th2 phenotype in ECA patients and it was consistent with increased level of Arg1, which was MDSCs and M2 associated cytokine. Recently, Gabitass et al. [24], reported that MDSCs were elevated in pancreatic and gastric cancer, and demonstrated they were an independent prognostic factors and associated with significant elevation of the Th2 cytokine IL-13. These results were partly consistent with our data. It should be mentioned that the IL-4 mRNA was increased in PBMC, while expression of IL-4 protein in plasma was unchanged in cancer patients. It was not an entirely consistent result, which might due to IL-4 gene transcription and its protein secretion were not synchronous, further research was necessary to investigate this in more detail.

Some researchers believe that IL-4 and IL-13 are enough to play important roles in M2 macrophages activation [6]. In addition, Gabitass RF et al. [24] and Pesce JT et al. [25], indicated that the Th2 cytokines: IL-4 and IL-13 can upregulate arginase activity thereby increasing the suppressive function of MDSCs and M2 macrophages. Therefore, we propose that IL-4, IL-13 and Arg1 are key factors mediating the distribution of MDSCs and M2 macrophages, and are closely related to Th2 cell polarization. Ostrand-Rosenberg et al. [26], show that cross-talk between MDSCs and macrophage promotes synergy among these cells thereby augments the immune suppressive effects of the individual cell populations. As a result, MDSCs and macrophages in the tumor microenvironment are inextricably interconnected so that the functions of one population are impacted by the quantity of the other populations [27]–[30]. In a recent review of survival analyses, it was found that many studies have indicated clinical and pathologic characteristics of patients with esophageal carcinoma as explanatory variables with respect to survival, and showed a better survival rate for women or young patients. The reasons for this are likely to include a combination of better general health and more effective immune state [31]–[32]. However, there were limitations with the present study in which the correlation between MDSCs% and the clinical pathological factors of ECA patients were not analyzed, it should be considered in our future work.

## Conclusion

We revealed an interesting connection between Th2 cells associated cytokines and MDSCs or M2 macrophages in ECA. The changes detected in the IL-4, IL-6, IL-13 and GATA3 levels correlated positively with MDSCs and M2 macrophages activation, but IFN-γ played the opposite effect. The close relationship between circulating MDSCs and tissue infiltration of M2 macrophages indicated that infiltration of M2 macrophages in ECA tissues might be related to MDSCs polarization involving Th2 advantage. These results provide better understanding of systemic immunosuppression such as the interaction between T cells and MDSCs or M2 macrophages, systemic cytokines and their signalling pathways, and may contribute to triggering new strategies for anticancer therapy, which are based on modulation of the immunosuppressive effects of tumor-associated MDSCs, M2 macrophages and Th2 cells.
